# *Anemoside B4* attenuates intestinal damage in chickens infected with *Eimeria tenella*: Mechanisms involving antioxidant defense, immune modulation, and barrier repair

**DOI:** 10.1016/j.psj.2025.106248

**Published:** 2025-12-10

**Authors:** Mohan Yang, Haixia Han, Zhe Zheng, Qi Xin, Baihui Zhang, Tingting Yu, Xuwen Wang, Yanchun Wang, Yanan Cai

**Affiliations:** College of Animal Science and Technology, Jilin Agricultural University, Changchun 130118, China

**Keywords:** *Anemoside B4*, *Eimeria tenella*, Inflammation, Oxidative stress

## Abstract

This study aimed to investigate the reparative effects and underlying mechanisms of *Anemoside B4* (AB4) on intestinal damage induced by *Eimeria tenella* (*E. tenella*) infection in chickens. Eighty 14-day-old broilers were allocated into four groups: control (CON), *E. tenella*-infected (*E. tenella*), AB4-treated (*E. tenella*+AB4), and diclazuril-treated (*E. tenella*+DC). Infected birds were orally inoculated with 4 × 10⁴ sporulated oocysts, followed by oral administration of AB4 (40 mg/kg BW) or Diclazuril (1 ml/bird, 0.2 mg/mL) at 12 hours post-infection. Key findings demonstrated that AB4 exhibited significantly superior radical scavenging activity (82.7–93.1 %) against superoxide anion, 2,2-diphenyl-1-picrylhydrazyl (DPPH), and hydroxyl radicals compared to Diclazuril (<16.3 %) . The Anticoccidial Index (ACI) for the AB4 group (163.05) indicated moderate efficacy and was comparable to the diclazuril group (161.69) (P>0.05), with significantly reduced cecal lesion scores. Concurrently, AB4 downregulated serum pro-inflammatory cytokines (IFN-γ, IL-6) and upregulated IL-10, enhanced the splenic CD4⁺/CD8⁺ ratio (with superior restoration to Diclazuril at D14, P<0.01), and promoted expression of tight junction genes (ZO-1, P<0.01; Occludin, P<0.05 at D7/D14), corroborated by histopathological evidence of intact cecal villus architecture. These results establish that AB4 effectively mitigates coccidial enteritis through a tripartite synergistic mechanism involving potent antioxidant activity, immune homeostasis restoration, and intestinal barrier repair, offering comparable efficacy to conventional anticoccidials with enhanced safety as an eco-friendly alternative.

## Introduction

Avian coccidiosis, a parasitic disease caused by Eimeria spp., poses a substantial threat to the global poultry industry, with annual economic losses estimated at approximately $3 billion (Blake & Tomley, 2014; Franzo et al., 2025). Among the various species, Eimeria tenella is particularly detrimental due to its tropism for cecal tissue, where its complex life cycle—involving sporozoite invasion, merogony, and gametogony —leads to severe hemorrhagic enteritis and significant morbidity (Shirley et al., 2005). Current control strategies predominantly rely on chemical anticoccidials and live vaccines (Dalloul & Lillehoj, 2006; Ritzi et al., 2016). However, the utility of these interventions is increasingly compromised by the widespread emergence of drug resistance—over 80 % of farms report reduced sensitivity to agents like diclazuril —and the limited cross-protective efficacy and potential virulence reversion associated with live vaccines. These challenges underscore the urgent need for novel and sustainable control alternatives.

Medicinal plants, with their multi-target mechanisms and favorable safety profiles, represent a promising source of such alternatives. *Pulsatilla chinensis*, a traditional herb used for dysentery, has garnered attention for its potent pharmacological activities, largely attributed to its bioactive saponins (Zhang et al., 2019). Among these, Anemoside B4 (AB4) has been identified as a key constituent. Previous studies, primarily in mammalian models of ulcerative colitis, have demonstrated that AB4 possesses remarkable antioxidant, anti-inflammatory, and immunomodulatory properties (Xu et al., 2023). Its mechanisms include scavenging free radicals (Zhang et al., 2019), regulating T-cell subsets and cytokine balance (Lillehoj et al., 2012), inhibiting pro-inflammatory cytokine release via suppression of the NF-κB pathway (Xu et al., 2023), and enhancing intestinal barrier function by upregulating tight junction proteins (Wu et al., 2025).

Despite these promising findings, critical gaps remain in understanding the potential of AB4 against avian coccidiosis. Specifically, its efficacy and mechanistic actions within the context of E. tenella infection are poorly defined. Key unanswered questions include: How does AB4 dynamically modulate the host immune response and inflammatory cascade post-infection (Hong et al., 2006). How does its performance compare quantitatively to conventional anticoccidial drugs regarding comprehensive metrics like the Anticoccidial Index (Giannenas et al., 2014). What are the precise signaling pathways involved in its protective effects in an avian host (Wallach et al., 1995)?

To address these questions, this study was designed to systematically investigate the therapeutic potential and molecular mechanisms of AB4 in chickens infected with E. tenella. We hypothesized that AB4 would ameliorate E. tenella-induced intestinal damage through a coordinated mechanism involving immunomodulation, anti-inflammation, and barrier repair. Our specific objectives were to: 1) dynamically assess its effects on splenic T-cell populations and systemic immune markers (Allen & Fetterer, 2002); 2) evaluate its capacity to regulate cecal inflammatory cytokine expression (Awad et al., 2018) and enhance barrier protein genes; and 3) determine its overall efficacy using the Anticoccidial Index (ACI) and histopathological scoring (Johnson & Reid, 1970). This work provides the first comprehensive evidence for a "tripartite synergistic mechanism" of AB4 in avian coccidiosis, establishing a scientific foundation for its development as a natural anticoccidial agent.

## Materials and methods

### Animals, parasites and drugs

Eighty 1-day-old commercial broiler chicks (a standard white-feathered line) were purchased from the Poultry Breeding Experimental Station of the Jilin Academy of Agricultural Sciences, Changchun, Jilin Province, China. *E. tenella* was provided by the Parasitology Laboratory, College of Veterinary Medicine, Jilin Agricultural University, China.

This study employed a completely randomized design (CRD). A power analysis was conducted prior to the experiment using G*Power software (version 3.1.9.7) to determine the minimum sample size required. With an effect size (f) of 0.4, an alpha error probability of 0.05, and a power (1-β) of 0.8, the analysis indicated that a minimum of 16 animals per group was necessary. To account for potential mortality and ensure robust statistical power, we included 20 chickens per group (n=20), resulting in a total of 80 animals.

All animal experimental procedures were approved by the Institutional Animal Care and Use Committee (IACUC) of Jilin Agricultural University (Approval No.:MOE22US2A20192027N) and were conducted in strict accordance with the institution's guidelines for the care and use of laboratory animals.

### Major reagents and instrumentation

Diclazuril solution (2.5 mg/mL) was purchased from Changchun Kangmu Veterinary Pharmacy Co., Ltd. (Changchun, China; Catalog No.: KM-20220915). Anemoside B4 (AB4) was provided by the Parasitology Laboratory, College of Veterinary Medicine, Jilin Agricultural University. The chicken splenic lymphocyte isolation kit (Bovine Serum Albumin Method) was obtained from Beijing Solarbio Science & Technology Co., Ltd. (Beijing, China; Catalog No.: P8880). Anti-chicken CD4-PE, CD3-FITC, and CD8-PE antibodies were sourced from the same laboratory. Chicken immunoglobulin A (IgA; Catalog No.: YX-070721Ch), interleukin-10 (IL-10; Catalog No.: YX-070721Ck), interferon-gamma (IFN-γ; Catalog No.: YX-070721Ci), and immunoglobulin Y (IgY; Catalog No.: YX-070721Cj) ELISA kits were purchased from Shanghai Youxuan Biotechnology Co., Ltd. (Shanghai, China). The PrimeScript™ RT Reagent Kit with gDNA Eraser (Catalog No.: RR047A), TB Green® Premix Ex Taq™ II (Tli RNaseH Plus) (Catalog No.: RR820A), and total RNA extraction reagent RNAiso Plus (Catalog No.: 9108) were acquired from TaKaRa Bio Inc. (Dalian, China). Superoxide anion assay kit (Catalog No.: A052-1-1), DPPH scavenging activity kit (Catalog No.: A015-1-1), and hydroxyl radical scavenging activity kit (Catalog No.: A018-1-1) were procured from Nanjing Jiancheng Bioengineering Institute (Nanjing, China).

### Antioxidant capacity assay and in vivo dose determination

The antioxidant activities of AB4 (0.5, 1.0, 2.0 mg/mL) and diclazuril (DC, 0.005, 0.01, 0.02 mg/mL) solutions were evaluated in vitro. The superoxide anion (O₂⁻) scavenging rate was assessed using a commercial kit: 100 μL of sample was mixed with 200 μL of reaction buffer, incubated at 37 °C for 30 min, followed by the addition of 50 μL of 1.25 nM nitrobule tetrazolium (NBT) for color development, and the absorbance was measured at 550 nm. The 2,2-diphenyl-1-picrylhydrazyl (DPPH) scavenging activity was determined by mixing 100 μL of sample with 100 μL of 0.1 nM DPPH ethanol solution, incubating in darkness at 25 °C for 20 min, and reading the absorbance at 517 nm. The hydroxyl radical scavenging rate was measured by combining 50 μL of sample with 100 μL of working solution, incubating at 37°C for 40 min, and measuring the absorbance at 510 nm. All scavenging rates were calculated as (1- A∼sample∼/A∼control∼) × 100 %. L-Ascorbic acid (0.1 mg/mL) served as the positive control, and 0.1 % dimethyl sulfoxide (DMSO) was used as the vehicle control for DC groups. All assays were performed in triplicate using a microplate reader.

Based on the in vitro results, the superoxide anion scavenging IC₅₀ of AB4 was determined to be 0.62 mg/mL, and the concentration required for 90 % hydroxyl radical clearance was 1.85 mg/mL. These effective concentrations (ECs) were subsequently used to calculate the in vivo dose for the chicken model via a pharmacodynamic translation model: Dose = [EC (mg/mL) × Vd (L/kg) × 1000 × κ] / F. In this formula, Vd (the volume of distribution) = 4.2 L/kg, F (the oral bioavailability) = 15 %, and κ = 5 (a safety factor accounting for compromise in the infection microenvironment). This calculation yielded an effective AB4 dose threshold of 38.6–46.2 mg/kg, which established 40 mg/kg as the benchmark therapeutic dose for daily oral administration.

### Experimental design and sample collection

Eighty 1-day-old broiler chickens were housed in disinfected cages. They were provided with a commercial pelleted diet that met all nutritional requirements and autoclaved water to prevent potential microbial contamination. On day 14, the chicks were fasted and weighed, then randomly divided into four groups (n=20 per group): Control group (CON group); *E. tenella*-infected group (*E. tenella* group); AB4-treated group (*E. tenella* + AB4 group); Diclazuril-treated group (*E. tenella* + DC group). Except for the CON group, all chicks were orally inoculated with 4 × 10⁴ sporulated *E. tenella* oocysts, while the control group received 1 ml Phosphate-buffered saline (PBS). At 12 hours post-challenge, chickens in the *E. tenella* + AB4 group received pulsatilla saponin B4 solution via daily oral gavage at 40 mg/kg body weight (BW). The dosage of AB4 (40 mg/kg BW) was selected based on both literature reference and preliminary experimental validation. It falls within the effective range (20–50 mg/kg) reported for Pulsatilla chinensis extracts in avian intestinal inflammation models (Wu et al., 2025; Zhang et al., 2019). Furthermore, a prior dose-ranging study in E. tenella-infected chicks confirmed that 40 mg/kg yielded the optimal anticoccidial efficacy and safety profile. In contrast, the *E. tenella* + DC group received daily oral administration of diclazuril solution (0.2 mg/mL) at 1 ml per chicken. Chickens were monitored daily for clinical signs and fecal consistency. On days 3, 7, and 14 post-infection, chickens were euthanized after a 12-hour fast (water allowed).

Before dissection, body weight was recorded, and blood samples were collected via cardiac puncture for ELISA analysis. The spleen was aseptically excised and weighed to calculate the spleen index (spleen weight/body weight). The cecum was collected to measure its length, and cecal lesion scores were determined according to the method of Johnson and Reid (1970) as follows: 0, no gross lesions; 1, small scattered petechiae or mild mucosal thickening; 2, moderate number of petechiae or luminal blood; 3, large amount of luminal blood with thickened walls; 4, severe hemorrhage, fecal cores, or death due to coccidiosis (Johnson & Reid et al., 1970). A portion of the cecal tissue was fixed in 4 % paraformaldehyde for histopathological examination, while the remaining samples were stored at -80°C for subsequent qPCR analysis.

### Determination of anticoccidial index (ACI)

Following euthanasia, the anticoccidial index (ACI) was calculated for each group using the formula: ACI = [Relative weight gain ( %) + Survival rate ( %)] – [Oocyst value + Lesion value], where: Relative weight gain ( %) = (Weight gain of infected-treated group / Weight gain of control group) × 100; Survival rate ( %) = (Number of surviving chickens / Total chickens per group) × 100; Oocyst value was assigned based on oocyst reduction ratio (Control OPG / Treated OPG × 100): < 1 % = 0, 1–25 % = 5, 25–50 % = 10, 51–75 % = 20, > 75 % = 40; Lesion value = Mean lesion score × 10 (lesion scored per Johnson & Reid (Allen, et al., 2002)); OPG (oocysts per gram) = Total oocyst count × 1000. Anticoccidial efficacy was classified as: Poor (ACI < 120), Moderate (120 ≤ ACI < 160), Good (160 ≤ ACI < 180), or Excellent (ACI ≥ 180) according to established criteria (Chapman, et al., 2010).

### ELISA analysis of serum cytokines

Serum samples collected were centrifuged at 3,000 × g for 15 min at 4°C. Levels of IgA, IL-10, IFN-γ, and IgY were quantified using commercial ELISA kits according to the manufacturer's protocols. Standard curves were generated by: Plotting absorbance (Y-axis) against standard concentrations (X-axis) in Microsoft Excel; Fitting a linear regression equation (y = ax + b, R² ≥ 0.99); Calculating sample concentrations by interpolating absorbance values into the regression equation.

### Histopathological examination of Cecal tissues

Cecal tissues were fixed in 4 % paraformaldehyde (4°C, 24 hr), processed through standard paraffin embedding (gradient ethanol dehydration → xylene clearing → paraffin infiltration), and sectioned at 4 μm thickness. Sections were stained with hematoxylin and eosin (H&E). Histopathological evaluation was performed using light microscopy (Nikon Eclipse E100, Japan) to assess: Villus architecture (atrophy/fusion/fragmentation); Crypt integrity (hyperplasia/dilatation/necrosis); Inflammatory infiltration (lymphocytes/macrophages/eosinophils); Submucosal hemorrhage and edema.

### Flow cytometric analysis of splenic T Lymphocyte Subsets

Splenic lymphocytes were isolated aseptically using a commercial isolation kit, stained with pre-diluted anti-chicken CD4-PE (clone CT-4) and CD8-FITC (clone CT-8) antibodies (20 × dilution in PBS) for 15 min at RT in the dark, washed twice with FACS buffer (2,000 × g, 4 °C, 5 min), and filtered through 70-μm mesh. Samples were analyzed on a BD Accuri C6 flow cytometer employing a three-step gating strategy: (1) FSC-A/SSC-A for viable lymphocytes, (2) FSC-H/FSC-A for single cells, and (3) FL1(530/30 nm)/FL2(585/40 nm) for T-cell subsets. Data were processed in FlowJo v10.8.1 to quantify CD4⁺ % (PE⁺), CD8⁺ % (FITC⁺), and CD4⁺/CD8⁺ ratio.

### Cytokine expression

Cecal tissues were homogenized in liquid nitrogen, and total RNA was extracted using 1 ml of RNAiso Plus reagent (Takara, Japan). First-strand cDNA was synthesized from 1 μg RNA using the PrimeScript™ RT Reagent Kit with gDNA Eraser (Takara, Japan) according to the manufacturer's protocol. Quantitative real-time PCR was performed with SYBR Green Premix Ex Taq™ (Takara, Japan) on a CFX96 Touch™ system (Bio-Rad) to analyze gene expression of: Pro-inflammatory cytokines: IL-1β, IL-6, IL-8 (CXCLi2), TNF-α; Anti-inflammatory cytokine: IL-10; Gut barrier markers: Occludin, ZO-1; Primer Design and Validation: All primers (Table 1) were designed with Beacon Designer 8.14 (Premier Biosoft), Synthesized by Sangon Biotech (Shanghai, China), Validated for specificity (single-peak melting curves) and efficiency (90–105 %). Data Analysis:β-actin served as the endogenous control; each sample was run in triplicate technical replicates, and relative expression was calculated via the 2^(-ΔΔCt) method.

**Table 1:** Primer sequeCONes used for qPCR in this study.

Gene	Sequences(5′-3′)
IL-1β	(F) : 5′-GCTCTACACGTTCAGCACCA-3′ (R) : 5′-TGTCCTCATCCTGGAGGAGT-3′
IL-6	(F) : 5′-CAAGGTGACGGAGGAGGAC-3′ (R) : 5′-TGGCGAGGAGGGATTTCT-3′
IL-8	(F) : 5′-ATGAACGGCAAGCTTGGA-3′ (R) : 5′-GCAGTGGGGGCCGCTTGG-3′
TNF-α	(F) : 5′-CAGGTGAGAGCGGTGGTG-3′ (R) : 5′-GGCAGGTGATGTGGGCTAC-3′
IL-10	(F) : 5′-TCACTTCCTCCTCCTCATCA-3′ (R) : 5′-GAGACGTTCGAGAAGATGGATG-3′
Occludin	(F) : 5′-CAGCAACAGCGTCTACCTCA-3′ (R) : 5′-GTCGTTGCCATAGCCATCAC-3′
ZO-1	(F) : 5′-ATGTCGCTGGAGACGAGTT-3′ (R) : 5′-CAGGTAGCCGTCCTTGTTGT-3′

### Data Analysis and Statistics

All data were processed and analyzed using GraphPad Prism software (version 9.0.0 for Windows, GraphPad Software, San Diego, California USA). The normality of data distribution was assessed using the Shapiro-Wilk test, and the homogeneity of variances was confirmed using Bartlett's test.

For comparisons across multiple groups at a single time point, a one-way analysis of variance (ANOVA) was employed, followed by Tukey's post-hoc test for multiple comparisons. This applies to data such as antioxidant capacity assays and ACI.

For data collected over multiple time points (e.g., cytokine levels, qPCR, flow cytometry), a two-way ANOVA was used with the factors being 'Treatment' and 'Time', followed by Sidak's multiple comparisons test to examine the effects of treatment at each specific time point.

Data are presented as the mean ± standard error of the mean (SEM) unless otherwise stated. A P-value of less than 0.05 (P < 0.05) was considered statistically significant. The specific statistical tests used for each Fig. are detailed in the corresponding Fig. legend. For the Anticoccidial Index (ACI), the Chi-square test was used to compare survival rates between groups.

## Results

### In Vitro antioxidant capacity assay of AB4 and DC

**Fig. 1:**
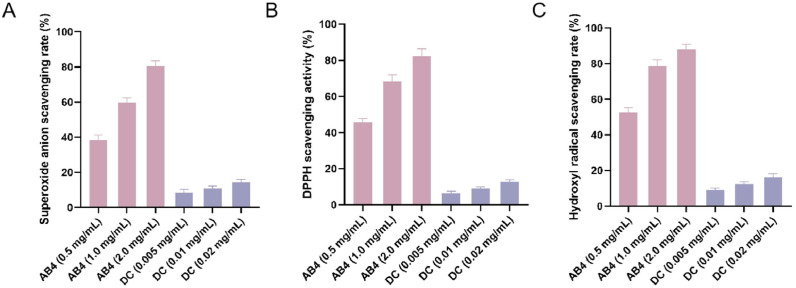
Results of in vitro antioxidant capacity assays for AB4 and DC. (A) Superoxide anion scavenging rate; (B) DPPH scavenging rate; (C) Hydroxyl radical scavenging rate.

As shown in Fig. 1, AB4 exhibited significant dose-dependent antioxidant activity (P<0.01) within the concentration range of 0.5–2.0 mg/mL, with superoxide anion, DPPH, and hydroxyl radical scavenging rates progressively increasing to 82.7–93.1 % at the highest concentration (2.0 mg/mL). In contrast, Diclazuril (DC) demonstrated consistently low scavenging rates (<16.3 %) for all three radicals at equivalent anticoccidial concentrations (0.005–0.02 mg/mL) without concentration-dependent effects (P>0.05). Comparative analysis revealed AB4 (2.0 mg/mL) significantly outperformed DC (0.02 mg/mL) in antioxidant efficacy (P<0.001), exhibiting 5.6–6.9-fold higher scavenging capacity. The analysis yielded an AB4 effective dose threshold of 38.6–46.2 mg/kg, establishing 40 mg/kg as the benchmark therapeutic dose for daily oral administration. For DC, 0.2 mg/mL solution was administered at 1 ml daily per bird according to standard anticoccidial regimens.

### Anticoccidial index (ACI) results in chickens from each groups

**Fig. 2:**
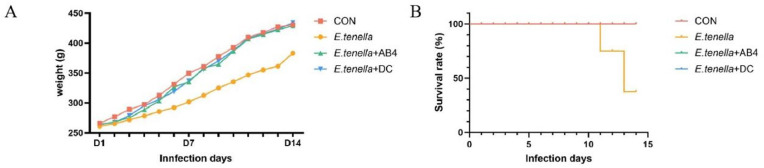
Growth Performance of Chickens:(A) Body Weight Change.(B) Survival Rate.

Initial and final body weights were recorded to calculate weight gain. Chickens in the E. tenella group exhibited clinical signs including lethargy and apparent anorexia by post-infection day 3 (D3). D7 observed characteristic bloody feces. Pathological examinations (D3/D7/D14) revealed: Markedly enlarged ceca with thickened walls—diffuse mucosal hemorrhage accompanied by retention of bloody loose stool. Two mortalities occurred at D11 and D13 (cumulative mortality: 20 %), with microscopic examination confirming *E. tenella* infection as the cause of death(Fig. 2**. B**). In contrast, the E. tenella + AB4 and E. tenella + DC groups exhibited only apparent transient decreases in feed consumption and lethargy at D3, based on clinical observation. These clinical signs gradually alleviated after D7. Compared to the CON group, no significant difference (P < 0.05) in weight gain was observed between *E. tenella* + AB4 and *E. tenella* + DC groups beyond D10 (Fig. 2**. A**). Gross pathology at all timepoints demonstrated: Intact cecal morphology, absence of mucosal hemorrhage, and standard luminal contents in the *E. tenella* + AB4 group. This group exhibited significantly lower lesion scores (P < 0.05) than the *E. tenella*-infected group, achieving an Anticoccidial Index (ACI) of 163.05 (Table 2). These results demonstrate that AB4 effectively mitigates clinical manifestations induced by *E. tenella* infection and provides moderate therapeutic efficacy against avian coccidiosis.

**Table 2:** Determination results of anti coccidiosis index in each group of chickens.

Groups	Survival Rate ( %)	Relative Weight Gain Rate ( %)	Value of oocysts	Lesion Scores	ACI
CON	100a	100a	0	0a	200a
*E. tenella*	90b	73.07±0.84d	40	40.48±1.19d	82.59±1.27d
*E. tenella*+AB4	100a	98.81±1.05c	10	25.76±0.98b	163.05±0.74b
*E. tenella*+DC	100a	99.22±1.37b	10	27.53±1.02c	161.69±0.88c

Different superscript letters within a column indicate statistically significant differences (p < 0.05 by Tukey's test), while shared superscript letters denote no significant difference (p > 0.05). This convention applies to all subsequent Fig.s/tables.

### Serum cytokine and immunoglobulin dynamics

Cardiac puncture was performed on chickens from each group at 3, 7, and 14 days post-infection (D3/D7/D14). ELISA quantified serum levels of IL-10, IFN-γ, IgY, and IgA. Results (Fig. 2) demonstrated that compared to the CON group, the *E. tenella*-infected group exhibited significantly elevated serum levels of IL-10, IFN-γ, IgY, and IgA (P < 0.05), with peak values observed at D7 followed by a decline at D14. In contrast, both the *E. tenella*+AB4 and *E. tenella*+DC groups showed significantly reduced levels of these markers relative to the *E. tenella* group (P < 0.05), progressively approaching CON group levels over time.

### Histopathological examination of Cecal tissues

Histopathological examination of cecal tissues collected from chicks at D3, D7, and D14 post-infection revealed distinct pathological changes following H&E staining. The CON group maintained standard cecal architecture with intact villi and no inflammatory cell infiltration throughout the observation period. In contrast, the *E. tenella* group exhibited progressively aggravated pathology: early villus sloughing and inflammatory infiltration were observed at D3; extensive villus necrosis/sloughing, marked hemorrhage, and severe inflammation developed by D7; these lesions persisted with intensified villus damage at D14 (Fig. 4**.**). *E. tenella*+AB4 and *E. tenella*+DC showed minimal pathology post-infection: cecal structure remained essentially normal with only occasional mild localized inflammation at D3; villus integrity was preserved with negligible hemorrhage and mild confined inflammation at D7; by D14, villus morphology was substantially restored to near-normal without hemorrhage, accompanied by significantly resolved inflammatory infiltration, ultimately approximating the pathological status of the uninfected CON group.

### Flow cytometric analysis of splenic CD4⁺ and CD8⁺ T lymphocytes

At day 3 (D3), the *E. tenella* group exhibited a significantly elevated ratio compared to the CON group (P < 0.05), while the *E. tenella*+AB4 group showed a ratio significantly higher than that of the *E. tenella*-infected group (P < 0.05). By day 7 (D7), the ratio in the *E. tenella*-infected group had decreased significantly relative to the CON group (P < 0.05); however, the AB4 intervention group (*E. tenella*+AB4) demonstrated a highly significant increase in its ratio compared to the contemporaneous *E. tenella*-infected group (P < 0.01). At day 14 (D14), the ratio in the *E. tenella* group remained significantly lower than that in the CON group (P < 0.05). In contrast, the *E. tenella*+AB4 group not only displayed a highly significant elevation in its ratio compared to the *E. tenella*-infected group (P < 0.01), but its ratio level also reverted to near-normal levels comparable to the CON group, demonstrating superior efficacy relative to the *E. tenella*+DC group in restoring immune homeostasis.

### qPCR analysis of inflammatory-related gene mRNA expression in Cecal tissues

**Fig. 3:**
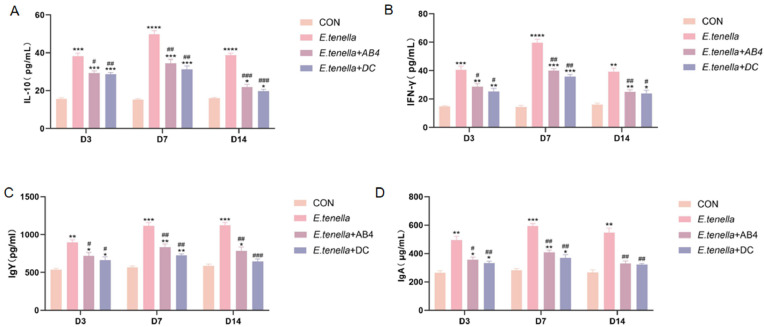
ELISA Results of Serum Cytokines of Chickens in each Group Statistical significance is denoted as follows: *p < 0.05, **p < 0.01, ***p < 0.001, ****p < 0.0001 vs. CON group; #p < 0.05, ##p < 0.01, ###p < 0.001 vs. *E. tenella* group. All subsequent Fig.s adhere to this convention unless otherwise specified.

Quantitative real-time PCR (qPCR) analysis of cecal tissue revealed AB4-mediated modulation of inflammatory and barrier gene expression following Eimeria tenella infection. Across all post-infection timepoints (D3, D7, D14), the *E. tenella*+AB4 group exhibited marked regulatory effects compared to the *E. tenella* group: 1) Pro-inflammatory cytokines (IL-1β, IL-6, IL-8, TNF-α) showed highly significant suppression of mRNA transcription (P < 0.0001), with expression reductions reaching extreme statistical significance (Fig. 6**A-D**); 2) The anti-inflammatory cytokine IL-10 demonstrated significant upregulation (P < 0.05), suggesting activation of immunoregulatory feedback (Fig. 6**E**); 3) For intestinal barrier genes, tight junction protein ZO-1 mRNA exhibited sustained and highly significant induction at all timepoints (P < 0.01, Fig. 6**G**), while occludin expression achieved significant elevation at D7 and D14 (P < 0.05, Fig. 6**F**) despite lacking statistical significance at D3.

## Discussion

**Fig. 4:**
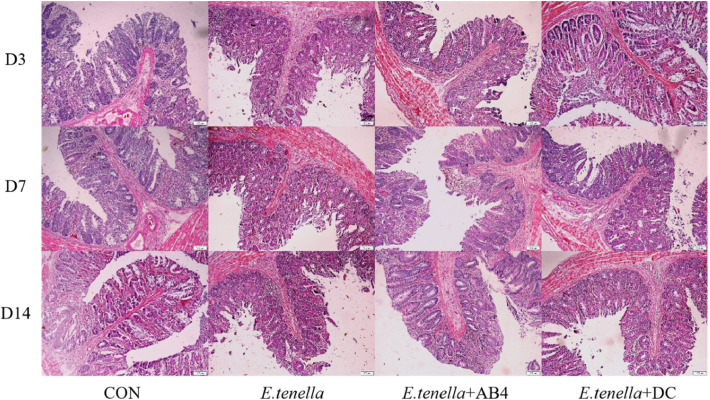
Observation of Cecal lesions of chickens in each group.

**Fig. 5:**
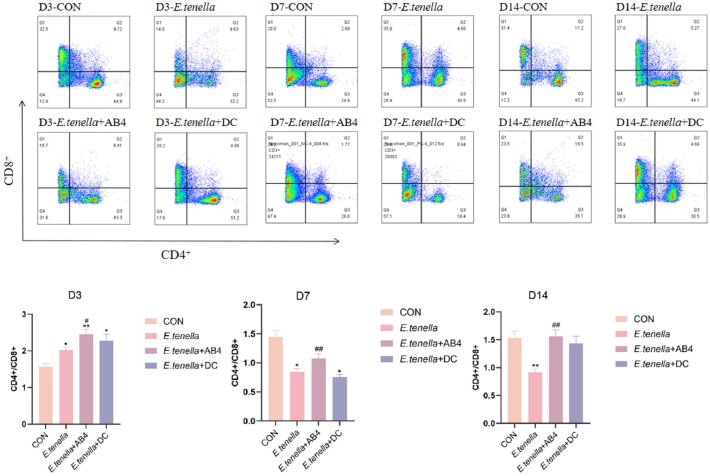
CD4+/CD8+T-cell Subsets distribution across experimental groups.

Note: *P < 0.05 vs. CON group;** P < 0.01 vs. CON group; #P < 0.05 vs. *E. tenella* group; ##P* < 0.01 vs. *E. tenella* group.

**Fig. 6:**
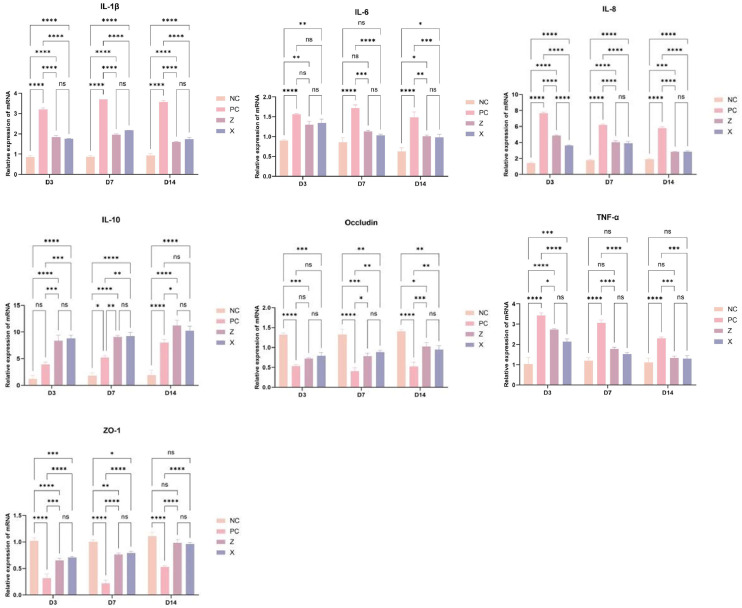
qPCR Analysis of mRNA Transcription Levels for Inflammation-Related Genes in Cecal Tissues Across Experimental Groups.

This study provides the first systematic elucidation of the reparative mechanisms of Anemoside B4 (AB4) against intestinal damage induced by Eimeria tenella infection in chickens. Our findings demonstrate that AB4 significantly alleviates the pathological progression of coccidiosis through a tripartite synergistic mechanism: antioxidant defense, immune homeostasis restoration, and intestinal barrier repair (Figs. 1**–**6). Its efficacy is comparable to that of the positive control drug diclazuril (DC), with distinct advantages in immunomodulation, positioning it as a promising multi-target therapeutic agent.

The potent antioxidant activity of AB4 constitutes a fundamental aspect of its therapeutic effect. Our in vitro assays confirmed its exceptional, dose-dependent radical scavenging capacity (Fig. 3). This property is critically important in coccidiosis, as Eimeria replication induces massive oxidative burst, leading to lipid peroxidation and cellular damage (Kim et al., 2020). The scavenging of superoxide anions by AB4 directly interrupts this vicious cycle, preserving enterocyte integrity. The observed activity is structurally attributed to the polyphenolic groups in its saponin backbone, which donate hydrogen atoms to stabilize free radicals (Zhang et al., 2019). Beyond direct scavenging, it is plausible that AB4, like other saponins, activates the Nrf2/Keap1 signaling pathway, a master regulator of the cellular antioxidant response, thereby upregulating endogenous enzymes like superoxide dismutase and heme oxygenase-1 (Surai et al., 2016). By mitigating the primary oxidative insult, AB4 creates a conducive microenvironment for subsequent repair processes.

The immunomodulatory prowess of AB4 was evidenced by the restoration of systemic and local immune homeostasis. The downregulation of pro-inflammatory cytokines (IFN-γ, IL-6) and concomitant upregulation of IL-10 (Fig. 3) indicates a shift from a damaging Th1-polarized response towards a regulatory phenotype. This is consistent with mechanistic studies showing that AB4 inhibits the PI3K/AKT/NF-κB axis, a central signaling hub for inflammatory gene transcription (Hu et al., 2025). More profoundly, the flow cytometric analysis revealed that AB4 normalized the splenic CD4⁺/CD8⁺ T-cell ratio (Fig. 5), which is often dysregulated during acute coccidial infection (Lillehoj et al., 2012). The superior restoration compared to DC by D14 suggests AB4 does not merely suppress immunity but actively recalibrates it. This aligns with reports that saponins can promote the differentiation of regulatory T (Treg) cells and modulate the TGF-β/IL-10 axis to suppress excessive Th1 activation without causing immunosuppression (Liu et al., 2021). This balanced immunomodulation is crucial for effective pathogen clearance while minimizing tissue damage.

A key finding of this study is the profound impact of AB4 on intestinal barrier integrity. The significant upregulation of tight junction genes ZO-1 and Occludin (Fig. 6**F–G**), corroborated by histopathological evidence of intact villus architecture (Fig. 4), underscores its reparative capacity. The disruption of tight junctions is a hallmark of E. tenella pathogenesis, facilitating parasite invasion and bacterial translocation. Our results suggest that AB4 directly targets this vulnerability. The mechanism may involve the activation of the AMPK/mTOR signaling pathway, which is known to be stimulated by certain saponins to enhance epithelial cell proliferation and junctional protein synthesis (Song et al., 2020). Furthermore, the modulation of serum IgA and IgY levels (Fig. 3) reinforces the "IgA-barrier cooperative defense" model, wherein secretory IgA helps to neutralize pathogens and toxins at the mucosal surface, working synergistically with a physically intact barrier to maintain gut homeostasis (Rothwell et al., 2004).

While AB4 and DC demonstrated comparable efficacy as measured by the Anticoccidial Index (ACI), AB4 presents several strategic advantages in the context of modern poultry production. First, as a plant-derived saponin, it circumvents the pervasive issue of drug resistance, which now affects over 87 % of farms using chemical anticoccidials like diclazuril (Peek et al., 2011). Second, its immunomodulatory properties, evidenced by the faster cytokine recovery and T-cell ratio normalization, suggest it can enhance immune memory and potentially confer longer-lasting resilience, a feature supported by findings that similar compounds promote CD8⁺ memory T-cell responses (Dalloul et al., 2005). Third, its natural origin generally correlates with a superior safety and residue profile, posing lower risks of hepatotoxicity and environmental impact compared to synthetic drugs (McDougald et al., 2013).

While this study establishes a solid foundation for AB4′s efficacy, several questions remain. Its direct anti-parasitic effect on the Eimeria life cycle, particularly on sporulation and merogony, needs verification through in vitro sporulation assays (Kogut et al., 2018). Furthermore, its efficacy against challenging mixed Eimeria species infections warrants investigation. Given the global push to restrict antibiotic and anticoccidial use, AB4 represents a viable, eco-friendly alternative. Future research should explore its potential in synergistic combinations, for instance, with probiotics or vaccines, to further enhance innate and adaptive anticoccidial immunity (Ritzi et al., 2016).

In summary, this research delineates a tripartite synergistic mechanism through which AB4 effectively repairs E. tenella-induced intestinal damage: potent direct and indirect antioxidant defense that quenches initial oxidative stress, balanced immune homeostasis restoration that recalibrates the host response without immunosuppression, and robust intestinal barrier repair that restores mucosal integrity. At a dosage of 40 mg/kg, AB4 achieves anticoccidial efficacy comparable to diclazuril, while offering the critical advantages of mitigating drug resistance risk, fostering immune memory, and exhibiting high safety, thereby positioning it as a promising natural alternative for integrated coccidiosis control.

## Funding

Key Research and Development Project of Jilin Provincial Department of Science and Technology, Authorization number 20220202062NC.

## CRediT authorship contribution statement

**Mohan Yang:** Writing – review & editing, Writing – original draft. **Haixia Han:** Investigation. **Zhe Zheng:** Resources. **Qi Xin:** Validation. **Baihui Zhang:** Formal analysis. **Tingting Yu:** Software, Formal analysis. **Xuwen Wang:** Visualization. **Yanchun Wang:** Funding acquisition. **Yanan Cai:** Resources, Funding acquisition.

## Disclosures

The authors declare no conflicts of interest.
